# Testing for HER2 in Breast Cancer: A Continuing Evolution

**DOI:** 10.4061/2011/903202

**Published:** 2010-12-06

**Authors:** Sejal Shah, Beiyun Chen

**Affiliations:** Department of Laboratory Medicine and Pathology, Mayo Clinic, Rochester, MN 55905, USA

## Abstract

Human epidermal growth factor receptor 2 (HER2) is an important prognostic and predictive factor in breast cancer. HER2 is overexpressed in approximately 15%–20% of invasive breast carcinomas and is associated with earlier recurrence, shortened disease free survival, and poor prognosis. Trastuzumab (Herceptin) a “humanized” monoclonal antibody targets the extracellular domain of HER2 and is widely used in the management of HER2 positive breast cancers. Accurate assessment of HER2 is thus critical in the management of breast cancer. The aim of this paper is to present a comprehensive review of HER2 with reference to its discovery and biology, clinical significance, prognostic value, targeted therapy, current and new testing modalities, and the interpretation guidelines and pitfalls.

## 1. Introduction and HER2 Biology

In 1981, Shih et al. discovered novel transmissible genes which caused transformation of NIH 3T3 cells upon transfection of DNA obtained from rat neuroblastomas [1]. Subsequently, the same group identified a 185,000 Dalton phosphoprotein obtained from the sera of young mice injected with secondary transfectants containing neuroblastoma transforming sequence [2]. This *neu *oncogene was later identified in genomes of fetal rat neuro/glioblastomas cell lines derived from tumors induced by ethylnitrosurea [3]. The nucleic acid sequence of the neu gene was homologous to the erb-B oncogene and the neu-associated tumor antigen p-185 was antigenically related but distinct from the epidermal growth factor (EGF) receptor. Two other groups by screening the human genomic library using v-erbB as screening probes independently isolated similar erb-B related genes HER2 [4] and c-erbB-2 [5]. Upon further analysis, neu, HER2, and c-erbB-2 were identified as identical genes mapping on the same chromosome location [4, 6]. In 1985, the amplification of this gene in DNA prepared from tissue of human mammary carcinoma was first demonstrated by King et al. [7]. 

HER2 is a member of the epidermal growth factor (EGF) receptor family which consists of four members: EGFR (HER1, erbB1), HER2 (erbB2), HER3 (erbB3), and HER4 (erbB4). The HER2 gene is located on chromosome 17q12 and encodes a 185-kDa protein product which is a transmembrane receptor protein with tyrosine kinase activity [8–10]. The receptor is structurally composed of an extracellular ligand-binding domain, transmembrane domain, and an intracellular tyrosine kinase catalytic domain. Upon activation by a ligand, the receptors dimerize and undergo transphosphorylation to activate various intracellular signaling pathways which mediate cell proliferation and differentiation [11, 12]. The cellular mechanism of HER2 activation is not completely understood, and there is no known stimulatory ligand for HER2 receptor homodimers. The HER2 receptor can, however, dimerize with other members of the EGFR family to form heterodimers, and these heterodimers involving HER2 have been shown to be more potent and stable [13]. In addition, crystal structures of rat HER2 have revealed a constitutively activated extracellular domain in the absence of a ligand [14].

Soon after its discovery, several in vivo and in vitro studies highlighted the oncogenic potential of HER2. Overexpression of HER2 was shown to be associated with cellular transformation and tumorogenesis in NIH 3T3 cells and human mammary epithelial cells [15–17]. In transgenic mice, overexpression of HER2 led to development of mammary tumors and induction of metastatic disease [18–20].

## 2. Prognostic Value of HER2

The prognostic value of HER2 amplification in human breast cancers was first determined by Slamon et al. in 1987 [21]. They evaluated tissues from 189 primary breast cancers and determined the role of HER2 as an independent prognostic factor. HER2 amplification was also shown to be a predictor of overall survival and time to relapse [21]. Currently, there are at least 107 published studies involving 39,730 patients that have discussed the prognostic significance of HER2 gene amplification (as assessed by southern blot, slot blot, polymerase chain reaction [PCR], fluorescent in situ hybridization [FISH] and chromogenic in situ hybridization [CISH]), and protein overexpression (as analyzed by western blot, immunohistochemistry [IHC], and enzyme-linked immunosorbent assay [ELISA]) [22]. Of these, 95 (88%) studies showed HER2 gene amplification or protein overexpression in breast cancer as an important predictive factor by either univariate or multivariate analysis. Multivariate analysis was performed on 93 studies of which 68 (73%) showed HER2 as an independent adverse prognostic factor. However, in 13 (12%) studies there was no correlation between prognosis and HER2 status [22]. 

In node-positive patients, HER2 amplification or protein overexpression has been shown to be a poor predictor of clinical outcome [21, 23–35]. A recent study by Gilcrease et al. has shown that any degree of HER2 overexpression (1+, 2+ or 3+) was associated with increased tumor recurrence and decreased patient survival in a node-positive cohort of breast cancer patients (*n* = 91) treated with doxorubicin-based chemotherapy without trastuzumab [36]. A different study showed a distinct, intermediate outcome in patients with low-level HER2 amplification by FISH, with a ratio of 1.5–2.2, compared to HER2 unamplified tumors and tumors with HER2 ratios greater than 2.2 [37].

The predictive value of HER2 in node negative patients has been contentious. While some studies verify the adverse predictive value in node negative patients [24, 32, 35, 38–47], others have found no significant correlation with clinical outcome [23, 25, 29–31, 48–51]. The differences in these study conclusions may be attributed to a multitude of factors including differences in the number of patients, patient population including those receiving systemic adjuvant therapy, length of followup, and most importantly HER2 status determination and interpretation techniques.

## 3. Predictive Value of HER2

In addition to the prognostic significance in breast cancer, HER2 amplification and protein expression has been shown to predict and modulate response of conventional chemotherapeutic agents. 

### 3.1. Combination Chemotherapy

Conflicting studies have been reported regarding the benefit of combination chemotherapy with cyclophosphamide, methotrexate, and fluorouracil (CMF) in HER2-positive tumors. Some studies have shown decreased responsiveness of HER2-positive tumors to CMF therapy. Gusterson et al. reported a randomized study involving 1,506 breast cancer patients enrolled in the international (Ludwig) breast cancer study group trial V [30]. The patients were divided into subgroups of lymph-node positive (*n* = 746) and lymph-node negative (*n* = 740) patients. The patients in the node-positive group were given prolonged chemotherapy or a single cycle of perioperative chemotherapy (PeCT), and patients in the node-negative group were given single cycle of PeCT or no chemotherapy. They concluded that for node-positive patients, the effect of prolonged CMF chemotherapy, and for node-negative patients, the effect of PeCT on disease-free survival, was greater in HER2-negative tumors when compared to HER2-positive tumors defined as focal or diffuse membrane positivity by IHC [30]. Similar results were shown in a subgroup of breast cancer patients (*n* = 179) with low-risk lesions without significant in situ component [43]. In this subgroup, the HER2-positive tumors (focal or diffuse membrane staining by IHC) showed significant decrease in disease-free survival at 5 years (40% versus 80%; *P* < .0001) and overall survival (*P* = .0001) compared to HER2 negative tumors [43]. 

In contrast to these observations, a controlled clinical trial involving 386 node positive breast cancer patients with a 20-year followup who received 12 monthly cycles of adjuvant CMF (*n* = 207) or no further treatment after radical mastectomy (*n* = 179) showed that both HER2-positive (intermediate or strong membrane staining by IHC) and HER2-negative tumors benefited from treatment which was assessed by relapse-free survival and cause specific survival compared to the untreated patients [52]. These findings were confirmed by other large randomized study which had a median followup of 28.5 years [53].

### 3.2. Anthracycline-Based Chemotherapy

Though some studies have indicated that patients with locally advanced HER2 overexpressing breast cancers receiving prolonged or high-dose anthracycline-based chemotherapy show no significant change in survival [54], treatment failure [55], and development of distant metastasis [56] when compared to HER2-negative patients, most studies show benefit of anthracycline-based chemotherapy in HER2-positive tumors. Of 1572 patients with lymph node-positive early breast cancer enrolled in Cancer and Leukemia Group B (CALGB) trial randomized to receive high, moderate, and low doses of cyclophosphamide, doxorubicin, and fluorouracil, 442 random tumor samples were obtained and assessed for HER2 expression by IHC [57]. The results indicated that patients with high HER2 expression (≥50%) who received high-dose chemotherapy had a significantly longer disease-free survival and overall survival as compared to the patients with no or low HER2 expression (<50%) [57]. Similar observations of improved response to high-dose anthracycline-based chemotherapy in HER2 amplified lymph node-positive breast cancers have also been shown by other studies [58, 59]. 

This was further confirmed in a recent large randomized study involving tissues from 710 premenopausal women with axillary lymph node-positive breast cancer where amplification of HER2 (HER2 to chromosome 17 ratio of ≥2) was associated with clinical responsiveness to anthracycline containing chemotherapy containing cyclophosphamide, epirubicin and fluorouracil (CEF) when assessed for relapse-free survival and overall survival compared to patients receiving CMF or tumors that lacked amplification of HER2 [60]. Anthracyclines are topoisomerase inhibitors, and the response to these agents in HER2-positive tumors is postulated to be due to coamplification of topoisomerase II *α* (topo2a) gene which is located close to the HER2 gene on chromosome 17 [61–63]. Studies have shown that amplification of topo2a occurs exclusively in presence of HER2 amplification and that in the majority of tumors, topo2a amplification correlates with topo2a overexpression [64].

### 3.3. Tamoxifen

Approximately 75% of all invasive breast carcinomas are positive for estrogen receptors (ER) or progesterone receptors (PR) [65]. Even though HER2-positive tumors show a significantly decreased expression of ER or PR in comparison to HER2-negative tumors, a substantial proportion still express ER or PR [66]. Patients with advanced breast cancer expressing hormone receptors (HR) show increased (70%–80%) response to Tamoxifen therapy, though overall up to 50% of HR-positive tumors will not benefit, and approximately 10% of HR-negative tumors will respond to treatment [67]. Experimental and clinical evidence particularly in advanced-stage cancer have suggested an association between HER2 overexpression and resistance to endocrine therapies in general [68–73]. In a recent prospective study of 516 consecutive stage I-II patients, clinical outcome after 5–10 years following tamoxifen-based adjuvant therapy was compared between HR-positive/HER2-positive subgroup (*n* = 51) and HR-positive/HER2-negative subgroup (*n* = 129) [74]. Cases were considered HER2-positive if membrane staining in >1% was identified in tumor cells. The study concluded that the disease-free survival and overall survival in patients receiving Tamoxifen alone or after chemotherapy was significantly lower in HR+/HER2+ group when compared to HR+/HER2− group [74]. In another retrospective study, node-negative breast cancer patients randomly assigned to 2-year adjuvant Tamoxifen or no further therapy were analyzed for HER2 protein overexpression by IHC [75]. After a median followup of 12 years, univariate analysis showed that adjuvant Tamoxifen significantly prolonged disease-free survival and overall survival in HER2-negative cases whereas it had no effect in HER2-positive cases (membrane staining in >10% cells) [75]. 

In contrast to the above, a randomized controlled trial of 282 patients with ER positive tumors treated with adjuvant oophorectomy and Tamoxifen were evaluated for HER2 protein expression [76]. Univariate analysis showed risk reduction for all treated patients in both HER2-positive (*n* = 73) and HER2-negative subgroups (*n* = 209) with a greater benefit in the HER2-positive group [76]. In another study by Berry et al., HER2 status in 651 ER-positive, node-positive patients was evaluated by three different methods (IHC, FISH, and PCR), and clinical outcome was evaluated after Tamoxifen therapy [77]. They concluded that the disease-free survival and overall survival in the patients receiving Tamoxifen was not influenced by the HER2 status of the tumors [77].

### 3.4. Taxanes

Paclitaxol (Taxol), one the first taxanes examined in clinical trials has been shown to be effective against many cancers considered refractory to conventional chemotherapy. Paclitaxol exerts its cytotoxic effect by inhibiting microtubule disassembly and promoting tubulin polymerization, thus disrupting cell division [78]. Though in vitro studies have demonstrated resistance to taxanes in transfected mammary cells overexpressing HER2 [79, 80], in vivo studies have shown contradictory results. Baselga et al. studied the sensitivity of taxanes in women with metastatic breast cancer [81]. The response rate for taxanes was significantly greater in HER2-positive tumors (65%) versus HER2-negative tumors (36%). This sensitivity remained even after controlling for confounding variables which correlated with HER2 overexpression [81]. Similar benefits from paclitaxel containing regimens have also been shown by other studies in patients with HER2 gene amplification or protein overexpression and metastatic breast cancer [82, 83]. Contrasting to these observations, a randomized study involving 474 women showed that the response rate and overall survival were not related to HER2 status, and there was a trend towards shorter median time to treatment failure among women with HER2-positive tumors [84].

## 4. HER2-Targeted Therapy

### 4.1. Discovery of Trastuzumab

The high incidence of HER2 gene amplification and protein expression in breast cancer and its prognostic and predictive value make HER2 an attractive target for development of therapeutic agents. In 1985, soon after the discovery of HER2, a monoclonal anti-p185 antibody was shown to revert neu-transformed NIH 3T3 cells into a nontransformed phenotype [85]. Monoclonal antibodies targeting the extracellular domain of HER2 were subsequently developed by several laboratories [86–88]. Several other in vitro studies have confirmed the antineoplastic properties of monoclonal antibodies directed against HER2 expressing tumor cells demonstrated by inhibition of anchorage-dependent growth [89, 90], monolayer tumor growth [91], and colonies in soft agar [91–93] or by sensitizing the HER2 overexpressing cells to tumor necrosis factor alpha [92]. In addition, in vivo studies of monoclonal antibodies directed against HER2 have also shown to inhibit tumor cell growth in transgenic mice [90, 93]. 

The use of these murine antibodies, however, is limited clinically due to the development of neutralizing human antibodies upon long-term use. To circumvent this dilemma, one of the most potent growth inhibitory anti-p185HER2, designated muMAb4D5 was humanized by gene conversion mutagenesis [91, 94]. This fusion gene (rhuMAb HER2) combined murine antigen-binding loops and human variable region framework residues and IgG1 constant domains. The product trastuzumab (Herceptin), a humanized monoclonal antibody specifically targeting the extracellular domain of the HER2 receptor, was launched in 1998 after approval by the US Food and Drug Administration (FDA). There are several proposed mechanisms of trastuzumab action including inhibition of HER2 shedding, inhibition of PI3K-AKT pathway, inhibition of cyclin E/cdk2 complex activity, attenuation of cell signaling, antibody-dependent cellular cytotoxicity, and inhibition of tumor angiogenesis [95, 96].

### 4.2. Efficacy and Safety of Trastuzumab

Following preclinical testing, the first clinical evidence of anti-HER2 targeted therapy was provided by phase II trials reported by Baselga et al. [97]. The study was performed in 46 patients with metastatic breast cancer with HER2 protein overexpression with at least 25% of tumor cells exhibiting membrane staining as measured by IHC. All patients were given single-agent therapy with trastuzumab. The overall response rate (complete and partial remission) in assessable patients (*n* = 43) was 11.6%. Additionally, 37% of the patients achieved minimal responses or stable disease. These results were confirmed by larger multinational clinical trial involving 222 women with HER2-positive metastatic breast carcinoma that had progressed after chemotherapy. After treatment with trastuzumab monotherapy, the overall response rate was 15% (8 complete and 26 partial responses) with a median duration of response of 9.1 months [98]. In another study by Vogel et al., trastuzumab was given as first-line treatment in 114 randomized HER2-positive breast cancer patients with metastatic disease [99]. The overall response rate in this group was 26%. More significantly, the response rate in tumors with 3+ staining by IHC (strong complete membrane staining in >10% tumor cells) was 35% compared to absence of response in tumors with 2+ staining (weak to moderate complete membrane staining in >10% tumor cells). The response rate in tumors with HER2 gene amplification by FISH was 34% compared to 7% in tumors that were negative by FISH [99]. 

Phase III trials were reported by Slamon et al., where 469 women with progressive metastatic HER2 positive breast cancers were randomly assigned into two groups [100]. The first group (*n* = 234) received standard chemotherapy alone, and the second group (*n* = 235) received standard chemotherapy plus trastuzumab. Patients who received chemotherapy with trastuzumab showed longer time to disease progression (median, 7.4 versus 4.6 months), higher rate of response (50% versus 32%), longer duration of response (median, 9.1 versus 6.1 months), lower rate of death at 1 year (22% versus 33%), longer survival (median, 25.1 versus 20.3 months), and a 20 percent decrease in risk of death [100]. Favorable clinical outcome was also noted when trastuzumab combined with Paclitaxel was administered after doxorubicin and cyclophosphamide to patients enrolled in National Surgical Adjuvant Breast and Bowel Project (NSABP) B-31 and the North Central Cancer Treatment Group (NCCTG) N9831 trials with surgically removed HER2-positive breast cancers [101]. Similar results have been reported by other phase III trials evaluating response of HER2-positive breast cancers treated with neoadjuvant chemotherapy and trastuzumab [102–104]. 

During the clinical trials of trastuzumab, it was observed that a small proportion of patients developed cardiotoxicity manifested as congestive heart failure, cardiomyopathy, and/or decrease in ejection fraction [98]. Occurrence of these unexpected adverse events prompted a retrospective review of all patients enrolled in seven phase II and III trials. The analysis revealed increased risk of developing cardiac dysfunction in patients receiving trastuzumab [105]. The severity was observed to be greatest in patients receiving trastuzumab with anthracycline and cyclophosphamide (27%), compared to those receiving trastuzumab and paclitaxel (13%) or trastuzumab alone (3%–7%). It was also noted that though the cardiac dysfunction was symptomatic in most patients (75%), standard treatment for congestive heart failure led to improvement in most patients (79%). Overall, it was concluded that in spite of these adverse effects treatment was justified in patients with metastatic breast cancer due to the improved overall survival following therapy [105].

### 4.3. Newer HER2-Targeting Drugs

Lapatinib (Tykerb) is a novel reversible dual inhibitor of HER2 and EGFR tyrosine kinases [106, 107]. The antitumorogenic properties of this drug was examined in human normal and tumor-derived cell lines by in vivo and in vitro studies [108] and in patients with advanced malignancies [109]. Lapatinib was approved by the FDA in 2007 for use in previously treated advanced metastatic breast cancers which overexpressed HER2 in combination with Capecitabine [110]. In a randomized phase III clinical trial, 324 women with previously treated locally advanced or metastatic HER2-positive breast cancer were assigned to receive Lapatinib with Capecitabine or Capecitabine alone [111]. The patients with combination therapy had 49 (30%) disease progression events compared to 72 (45%) events with monotherapy. Additionally, the median time to progression was 8.4 months for patients receiving combination therapy compared to 4.4 months in patients receiving monotherapy [111]. In a similar phase III trial of 399 women, addition of Lapatinib showed prolongation of time to progression and a trend towards improved overall survival [112]. 

Other HER2-targeting agents which are still being developed and are in preclinical testing stages include Pertuzumab (Omnitarg), which binds HER2 and sterically hinders the recruitment of HER2 into heterodimers [113], and Ertumaxomab, a bispecific antibody targeting HER2 and CD3 [114]. Targeted therapy with MDX-H210 [115] and 2B1 [116] have shown limited response in initial clinical trials.

## 5. 2007 ASCO Update of HER2 as Marker for Breast Cancer

The American Society of Clinical Oncology (ASCO) published an update of recommendations for use of HER2 as a marker for breast cancer [117]. According to these updated guidelines, HER2 should be evaluated in every primary invasive breast cancer either at the time of diagnosis or at recurrence in order to guide selection of trastuzumab for treatment. Recommendations were also made regarding utility of HER2 to predict response to specific chemotherapeutic agents. It was suggested that if chemotherapy was considered in a patient with HER2-positive breast cancer, an anthracycline should be considered. For trastuzumab-based therapy, it was suggested that a nonanthracycline regimen may produce similar outcome. The benefit of taxane-based chemotherapy was considered controversial, and use of HER2 to guide its use was not recommended.

## 6. HER2 Testing

The importance of HER2 as a prognostic, predictive, and therapeutic marker in invasive breast cancer is well recognized, and therefore, it is critical to validate and standardize testing techniques in order to make an accurate assessment of HER2 status. The significant contradictions in various studies can at least in part be attributed to differences in HER2 testing and interpretation [118–121]. Techniques which have been used to assess HER2 protein overexpression are immunohistochemistry, ELISA analysis of tumor cytosols or serum, and Western blot, and methods used to evaluate HER2 gene amplification include Southern blot, slot blot, CISH, FISH, and PCR [22].

Use of solid matrix blotting techniques like Southern blot, slot blot, and especially Western blot are significantly limited due to the dilutional artifacts in the tumor sample. In breast cancer specimens, these artifacts may be composed of benign breast ductal cells, acini, stromal cells, inflammatory cells, and vascular structures resulting in false negative cases [120–122]. Additionally, false positive results may be obtained due to inclusion of in situ carcinoma which can express high levels of HER2 [123–126]. In addition, these techniques need a large amount of tissues which would not be available in biopsy specimens. PCR is a sensitive technique; however, it is also affected by dilutional artifacts, and the analysis is time consuming and labor intensive [120]. The absence of simultaneous morphological assessment in the above studies is also a significant disadvantage. 

Contrary to the above, analysis by IHC and FISH can be automated and allow the simultaneous assessment of tumor morphology while eliminating difficulties with dilution artifacts.

### 6.1. HER2 Immunohistochemistry

IHC analysis of HER2 is a simple-to-perform, widely available and inexpensive test. It is nevertheless affected by several variables including tissue-fixation methods, reagents, assay protocols, antibody sensitivities and specificities, and scoring systems [118, 127–129]. In general, testing of freshly frozen tissues is more reliable than paraffin-embedded tissues as formaldehyde causes cross linking of proteins hindering the access of antibody to the epitope [118, 122, 130]. However, practically, it is not possible to have fresh tissues available in all cases especially when testing at reference laboratories and analyzing archival tissues. 

The reagents and antibodies used in an assay are other critical factors. Antibodies differ in their sensitivity to detect HER2 epitopes. The important considerations in an assay are the type of antibody used, clonality of the antibody (monoclonal versus polyclonal), and the dilution factor used. Studies comparing different antibodies have shown marked variation in HER2 detection [118, 131, 132]. Press et al. conducted a study analyzing sensitivity and specificity of 7 polyclonal and 21 monoclonal anti-HER2 antibodies on paraffin-embedded tissues of 187 breast cancers with known HER2 protein overexpression and gene amplification analyzed by Northern blot, Western blot, IHC, and Southern blot performed on frozen tumor specimens [118]. The sensitivity of the antibodies ranged from 6% to 80% and none of the antibodies were able to detect all the cases of breast cancer with HER2 overexpression. In a recent study with the help of College of American Pathologists (CAP), HER2 proficiency was evaluated with use of HER2 peptide analyte controls. Of the 109 participants, who returned evaluable stained slides, suboptimal staining was identified in 20 (18.3%) cases. The causes of failure in these cases were antigen retrieval errors (35%), antibody or staining protocol problems (20%), or a combination of both (45%) [133]. 

Several studies have shown correlation between membrane-staining pattern of HER2 and protein overexpression [118]. Though cytoplasmic staining can be recognized in cases of breast cancer, it has not been shown to correlate with gene amplification [118], HER2 mRNA levels [134, 135], or have an association with poor prognosis in a subset of node-positive women [34]. One study, however, has shown an association between moderate to strong cytoplasmic staining of HER2 with poor prognosis [136]. Another limitation of IHC scoring system is interobserver variability, particularly in cases with moderate (2+) membrane staining [137, 138].

The two FDA-approved IHC-based tests for testing HER2 overexpression are HercepTest (Dako, Carpinteria, CA) which uses A085 polyclonal antibody and Pathway (Ventana, Tucson, AZ) which uses 4B5 monoclonal antibody. The overall concordance between DAKO HercepTest and clinical trial assays (CTA) in 548 breast tumor specimens was 79% [139]. However, a 2+ score by HercepTest did not correlate well with the CTA, where approximately 42% of cases with HercepTest 2+ score were negative by CTA (0–1+) [139]. The low specificity of HercepTest was also highlighted by other studies [132, 140]. The Pathway kit was first introduced in 2002 when it used a monoclonal antibody CB11. This antibody was replaced by a new monoclonal antibody 4B5 in 2008, which showed sharper membrane staining and less background staining when compared to CB11 and a higher correlation with FISH with an excellent interlaboratory reproducibility when evaluated in a total of 322 breast cancer patients [141].

### 6.2. HER2 Fluorescent In Situ Hybridization

FISH is a more reliable, reproducible, sensitive, and accurate procedure which is less affected by tissue fixation and analytical variables compared to IHC. It also offers the benefit of simultaneous evaluation of morphology and gene amplification. Relative to solid matrix blotting procedures, analysis of HER2 gene amplification by FISH showed a sensitivity of 98% and specificity of 100% [142]. The technique, however, is more complex and labor intensive than IHC. 

The FDA-approved FISH-based tests for HER2 amplification are PathVysion (Abbott Molecular, Des Plaines, IL), INFORM (Ventana, Tucson, AZ) and HER2 FISH pharmDx (Dako, Carpinteria, CA). The PathVysion HER2 probe kit is a dual color FISH (D-FISH) assay which uses probes targeting HER2 gene and chromosome 17 centromere. The HER2 gene amplification is calculated based on the ratio of HER2 gene copies per chromosome 17 copy number. On the other hand, the INFORM assay is a single-color FISH (S-FISH) assay with a HER2 probe alone. In this assay, the HER2 gene amplification is calculated as an absolute value of HER2 gene copy number per tumor nucleus. 

Several studies have assessed the use of tissue microarrays as an efficient method to analyze HER2 gene amplification by FISH in a high-throughput manner [143–145].

### 6.3. Concordance between FISH and IHC

In general, there is concordance between tumors scored as 3+ by IHC and FISH, while cases scored 2+ by IHC showed the most discrepancy [146–154]. Correlation studies in 2279 cases with invasive breast carcinoma showed a concordance of HER2 status between IHC and both D-FISH (87%) and S-FISH (86%) [155]. Specifically, excellent concordance was seen in groups scored 0, 1+, and 3+ by IHC for both D-FISH (97%) and S-FISH (96%), while the most discordant category was the group scored 2+ [155]. 

In a multicenter study involving 426 women with breast carcinoma being considered for trastuzumab study, the correlation of IHC by HercepTest and FISH by PathVysion was analyzed [156]. It was found that only 2/270 (0.7%) of IHC 0 or 1+ cases were FISH positive and 6/102 (5.9%) IHC 3+ cases were FISH negative. Of the 54 cases with 2+ staining, only 26 (48%) showed HER2 gene amplification by FISH [156]. Several other studies have also shown absence of gene amplification in subset of cases which were scored 2+ by IHC [147, 149, 153, 154, 157, 158]. Hence, a combined approach with IHC and FISH analysis was recommended for accurate HER2 testing particularly for cases with moderate staining with IHC [137, 148, 149, 152, 157].

In a study evaluating clinical outcomes of 799 patients enrolled in 3 clinical trials with 2+ and 3+ scoring on IHC, it appeared that clinical benefit from trastuzumab therapy was restricted to patients with FISH positive (78%) metastatic breast cancers with higher overall response rate and longer duration of survival when compared to FISH negative (22%) patients [159]. Hence, they concluded that analysis by FISH is a preferred method to select patients for trastuzumab therapy [159]. Other studies have also suggested the use of FISH as a superior method which should be done as the first line of HER2 status assessment [160–162] or at least in all cases scored 2+ or 3+ by IHC [163, 164]. In contrast to the above, an analysis of 2963 breast cancer specimens obtained from 135 hospitals and cancer centers showed that the FISH test had a significantly higher failure rate (5% versus 0.08%), reagent cost ($140 versus $10), longer testing time (36 hours versus 4 hours), and interpretation time (7 minutes versus 45 seconds) in comparison to IHC testing [165]. It was concluded that HER2 status determination is most effective by using IHC as the methods of choice and performing FISH in cases with moderate (2+) staining [165].

## 7. Current Issues with HER2 Testing

Several studies have identified a subset of false positive breast cancers that are IHC 3+ and negative by FISH ranging from 3% to 22% of all positive cases [146, 148, 153, 156–158, 160]. These inconsistencies may be due to several causes including variability in tissue fixation and processing, intratumoral heterogeneity, and polysomy of chromosome 17 [166, 167].

### 7.1. Effect of Polysomy 17 on HER2 Testing

Polysomy of chromosome 17 is frequent, and depending on the definition of polysomy, it may be seen in 20%–30% of invasive breast carcinomas [168–171]. Analysis of polysomy 17 requires the use of dual color FISH, and its presence can complicate accurate assessment of HER2 status [172]. Studies have shown polysomy 17 as a contributing factor in a small subset of tumors, which were IHC3+ but lacked HER2 gene amplification [166, 169, 171]. 

While some studies have shown an association between unamplified polysomy 17 tumors with IHC 3+ protein expression and adverse prognostic features [173], these observations have not been validated by others [170, 174]. A study by Hofman et al. reported a response to trastuzumab monotherapy in FISH-negative tumors with polysomy 17 [175]. However, in a recent study involving 405 patients with metastatic breast cancer, it was observed that polysomy 17 in absence of HER2 amplification did not predict the response to Lapatinib with Paclitaxol compared to paclitaxel alone [176]. 

A recent analysis of HER2 status by array comparative genomic hybridization in breast carcinoma samples (*n* = 97) has shown that polysomy 17 is a rare event and suggest that the cases detected by FISH represent amplification of chromosome 17 centromere rather than true polysomy [177].

### 7.2. Intratumoral Heterogeneity

Another pitfall in accurate HER2 status determination and discordance between FISH and IHC is the presence of intratumoral heterogeneity. Several studies have reported the presence of intratumoral heterogeneity of HER2 in breast cancers [178–181], which may reflect genetic divergence in the tumor cells during clonal evolution [182]. Intratumoral heterogeneity can also contribute to discordance in results between primary and asynchronous metastatic and recurrent tumors [180, 183], synchronous metastatic tumors [184] and small biopsy specimens [180, 181]. 

A study analyzing HER2 protein expression in patients with locally advanced breast cancers who received neoadjuvant chemotherapy (*n* = 39) and patients who did not receive chemotherapy (*n* = 60) reported that the HER2 IHC scores significantly reduced in patients who received therapy (28.5%) compared to those who did not (11.7%) [185]. In contrast, examination of HER2 amplification in needle core biopsies and subsequent excisions of 100 patients showed excellent concordance, even in a subset of patients who received neoadjuvant therapy, suggesting that heterogeneity is not a significant confounding factor when analyzing HER2 by FISH [186]. 

In 2008, the CAP/American College of Medical Genetics Cytogenetics resource committee panel defined and provided practice guidelines for breast tumors with genetic heterogeneity [187]. Genetic heterogeneity of HER2 is defined as presence of greater than 5% but less than 50% of infiltrating tumor cells with a HER2/CEP17 ratio of greater than 2.2 [187]. Currently, the clinical significance of genetic heterogeneity and possible benefit from anti-HER2 therapy is not known and additional clinical trials are required.

## 8. Newer Modalities of HER2 Testing

### 8.1. Chromogenic In Situ Hybridization

In 2008, FDA-approved SPOT-Light HER2 CISH assay (Invitrogen, Carlsbad, CA) which uses formalin-fixed paraffin-embedded sections and can be used to detect HER2 as a primary test or as a reflex test in IHC equivocal (2+) cases. Amplification by this method is defined as HER2 gene enumerated as greater than 5 dots, clusters (small or large), or a combination per nucleus in a majority (>50%) of carcinoma cells [188]. This is further categorized into low and high amplification. Nonamplification is defined as 1–5 dots of HER2 gene per nucleus present in a majority (>50%) of carcinoma cells [188].

Tanner et al. first described the utility of CISH as an alternative to FISH [189]. A high concordance between FISH and CISH has been established by several other studies [190–194]. In a recent study involving 226 consecutive cases of invasive breast carcinomas obtained from two institutions, tissues were evaluated for HER2 protein expression and amplification by IHC (HercepTest), FISH (PathVysion), and CISH (SPOT-Light) [195]. They compared the results between FISH and CISH using the manufacturer's criteria (nonamplified and amplified) and the ASCO/CAP criteria (nonamplified, equivocal, and amplified). The concordance between CISH and FISH for positive and negative results was 98.5% and 98.6% at the two institutions using the manufacturers' criteria and 99% and 99.1% using the ASCO/CAP criteria [195]. The advantages of CISH include ability to analyze the test by light microscopy, preservation of morphologic features, permanent signals which will not fade with slide storage, lower reagent costs, and need for less expertise than FISH [193, 196].

### 8.2. Metallographic In Situ Hybridization

Silver In Situ Hybridization (SISH) is an automated enzymatic metallographic ISH technique that is based upon deposition of silver at the target site following an enzymatic reaction. The signals are permanent and can be assessed by bright field microscopes. In a multi-institution study of 298 invasive breast carcinomas, concordance between HER2 gene amplification by SISH and FISH was 96.6% when analyzed by FDA approved criteria and 98.9% when analyzed by ASCO/CAP guidelines after excluding equivocal cases [197]. In addition, the study showed high interobserver reproducibility. Other studies have also shown SISH to be an accurate method to detect gene amplification in paraffin-embedded formalin-fixed tissue [198, 199] and cytology preparations [200]. 

Other bright field metallographic techniques which have been studied for analyses of HER2 status include gold-facilitated in situ hybridization [201] and EnzMet GenePro which allows simultaneous detection of HER2 gene status by deposition of silver and protein expression [202].

### 8.3. Brightfield Double In Situ Hybridization

Brightfield Double In Situ Hybridization (BDISH) is a recently described automated technique which utilizes two probes targeting HER2 gene and chromosome 17 centromere (CEN 17) and allows simultaneous analysis of morphological features by a brightfield microscope [203]. Their analysis of 94 breast cancer cases demonstrated a high concordance between HER2 FISH and BDISH using the historical scoring method (98.9%) and the ASCO/CAP criteria including the FISH equivocal cases (95.7%) and after excluding the FISH equivocal cases (100%) [203].

## 9. Current ASCO/CAP Guidelines for HER2 Testing and Interpretation

Accurate assessment of HER2 status is critical in management of patients with invasive breast cancer. In an attempt to standardize HER2 testing and to improve the accuracy and reproducibility of the test results, the American Society of Clinical Oncology/College of American Pathologists (ASCO/CAP) panel has made recommendations for HER2 interpretation and testing [204]. The panel recommended determination of HER2 status in all cases of invasive breast carcinoma. Algorithms for interpreting HER2 gene amplification by FISH and protein expression by IHC are provided. The guidelines by ASCO/CAP define an HER2 IHC staining of 3+ as uniform intense membrane staining in >30% of invasive tumor cells as compared to previously defined >10% strong staining. Cases with weak to moderate complete membrane staining in at least 10% of cells are considered equivocal (2+), and in these cases, HER2 gene amplification with fluorescent in situ hybridization (FISH) should be tested. For FISH, the tumor is negative for HER2 gene amplification if the ratio of HER2 gene signals to chromosome17 signals is <1.8 or HER2 gene copy number is <4.0, equivocal when the ratio is 1.8–2.2 or HER2 gene copy number is 4.0–6.0 and positive if the ratio is >2.2 or HER2 gene copy number is >6.0. Guidelines for tissue processing include keeping the time from tissue acquisition to fixation as short as possible and fixation in 10% neutral buffered formalin for 6–48 hours. Additional guidelines for optimal test validation, internal quality assurance procedures, external proficiency assessment, and laboratory accreditation are also provided. 

### 9.1. Impact of New ASCO/CAP Guidelines

Studies analyzing the impact of the new ASCO/CAP guidelines have shown an improved concordance between IHC and FISH results, improved accuracy, and decrease in number of inconclusive FISH tests after raising the cutoff level to greater than 30% invasive tumor cells for HER2 3+ tumors [205–207]. Other studies have additionally shown decrease in interobserver variability by application of the new criteria [208]. In another study, however, there was no change in concordance between FISH results and IHC3+ cases and all the 27 cases scored as 3+ by IHC remained 3+ after using the new threshold [209]. In our retrospective study, 12 (8.5%) of 141 cases had 11%–30% of invasive tumor cells with intense membrane staining which would have their status changed from 3+ to 2+ (equivocal) based on the new guidelines [210]. The overall concordance between FISH and IHC was improved; however, up to 3% of patients would be disallowed from receiving anti-HER2 therapy based on the new guidelines. Thus, the important question remains whether improved concordance translates into better prediction of response to anti-HER2 therapy. This is also critical in light of recent data, which demonstrated benefit of trastuzumab in patients with HER2 overexpression (IHC 3+) regardless of whether there was evidence of gene amplification [211, 212]. A retrospective analysis of 2268 patients from N9831 adjuvant trastuzumab phase III trial where enrollment was based on previous criteria of HER2 IHC > 10% (3+) or FISH ≥ 2.0 showed that a small percentage (1.5%) of patients eligible for trastuzumab therapy under FDA-approved definitions would not be eligible by the new ASCO/CAP guidelines. Additionally, the trastuzumab effect appeared similar for HER2-positive patients regardless of ASCO/CAP or FDA-approved guidelines [213].

## 10. Conclusions

In conclusion, the confirmed clinical advantages of HER2-targeted therapy in patients with HER2-positive disease necessitate that all patients continue to be tested for HER2 status on diagnosis [204, 211]. When conducting HER2 testing, we should be aware of various analytical and clinical factors that may affect the testing results and the clinical significance of false positive or negative results.
